# Longitudinal Changes in the Composition of the Penile Microbiome Are Associated With Circumcision Status, HIV and HSV-2 Status, Sexual Practices, and Female Partner Microbiome Composition

**DOI:** 10.3389/fcimb.2022.916437

**Published:** 2022-07-05

**Authors:** Supriya D. Mehta, Debarghya Nandi, Walter Agingu, Stefan J. Green, Fredrick O. Otieno, Dulal K. Bhaumik, Robert C. Bailey

**Affiliations:** ^1^ Department of Medicine, Division of Infectious Diseases, College of Medicine, Rush University, Chicago, IL, United States; ^2^ Division of Epidemiology and Biostatistics, School of Public Health, University of Illinois, Chicago, IL, United States; ^3^ Nyanza Reproductive Health Society, Kisumu, Kenya; ^4^ Genomics and Microbiome Core Facility, College of Medicine, Rush University, Chicago, IL, United States

**Keywords:** penile microbiome, HSV - 2, HIV, vaginal microbiome, bacterial vaginosis

## Abstract

**Background:**

Penile microbiome composition has been associated with HSV-2 and HIV in men and with bacterial vaginosis (BV) and HSV-2 in female sex partners. This study sought to 1) characterize penile microbiome composition over a 1-year period and 2) identify factors associated with penile microbiome composition over time.

**Methods:**

This prospective study of community-recruited heterosexual couples in Kenya measured penile and vaginal microbiomes *via* 16S ribosomal RNA gene amplicon sequencing at 4 time points over 1 year (1, 6, and 12 months after baseline). We used longitudinal mixed-effects modeling to assess associated demographic, behavioral, and disease factors and changes in community type, meatal taxa with the highest mean relative abundance, and alpha and beta diversity measures. We estimated group-based trajectories to elucidate compositional trends.

**Results:**

Among 218 men with 740 observations, men had a median age of 26 years, 11.6% were living with HIV, and 46.1% were HSV-2 seropositive. We identified 7 penile community types that varied with circumcision status, female partner vaginal microbiome community state type (CST), condom use, and penile washing. Across varying analytic approaches, 50%–60% of men had stable penile microbiome compositions. Alpha diversity measures were lower for circumcised men and those who reported condom use; they were stable over time but higher if female partners had diverse CSTs or BV. BV was positively associated with the relative abundance of numerous individual penile taxa. The decreased Bray–Curtis similarity was more common for men with HSV-2, and HSV-2 was also associated with a lower relative abundance of *Corynebacterium* and *Staphylococcus*.

**Conclusions:**

Over a 1-year period, penile microbiome composition was stable for a substantial proportion of men and was influenced by men’s circumcision status, sexual practices, female partner’s vaginal CST and BV status, and men’s HSV-2 status. In the female genital tract, a diverse CST is often associated with poorer health outcomes. Our results contribute toward understanding whether this framework extends to the penile microbiome and whether diversity and the associated penile microbiome compositions influence susceptibility or resilience to poorer health outcomes in men. Focusing on understanding how these factors influence the penile microbiome may lead to therapeutic avenues for reduced HSV-2 and BV infections in men and their female sex partners.

## Introduction

Randomized controlled trials demonstrated that voluntary medical male circumcision reduces penile anaerobic bacteria (Price et al., 2010; Liu et al., 2013), and this is a potential mechanism by which HIV risk is reduced (Liu et al., 2017). Subsequent studies demonstrate that the penile microbiome is associated with bacterial vaginosis (BV) (Eren et al., 2011; Zozaya et al., 2016; Mehta et al., 2020) and HSV-2 (Mehta et al., 2020) in female sex partners and with human papillomavirus and HIV (Onywera et al., 2020a) infections in men. Despite the potential contribution of the penile microbiome to sexual health outcomes in men and their female sex partners’ risk of BV and HSV-2, there is a limited description of the penile microbiome over time and factors associated with the longitudinal variation. Most studies have been cross-sectional or focused primarily on the role of circumcision status and have a short duration. We identified only one non-interventional, longitudinal study of the penile microbiome in the peer-reviewed literature. In a U.S. sample of 18 male adolescent participants aged 14–17 years (5 circumcised and 10 having had vaginal sex), Nelson et al. observed that several taxa with the highest mean relative abundance (RA) recovered from the coronal sulcus were stable across four measurements taken monthly over a 3-month period (Nelson et al., 2012).

In our analysis of 168 heterosexual couples in Kenya, we observed that baseline penile meatus microbiome composition predicted incident BV in female partners with high accuracy, up to 6 to 12 months after penile microbiome measure (Mehta et al., 2020). We posited that one explanation for the prediction’s durability over many months may be due to the temporal stability of the penile microbiome. The goals of this analysis were to 1) characterize penile microbiome composition over a 1-year period and 2) identify factors associated with penile microbiome composition over time. Given the interrelatedness of the penile microbiome and vaginal microbiome (VMB) in relation to several sexual and reproductive health outcomes, such longitudinal studies can yield value to identify epidemiological associations that may lead to improved sexual health outcomes for men and their female sex partners.

## Materials and Methods

This study was approved by the Institutional Review Board of the University of Chicago, IL, USA (2013-0511) and the Ethical Review Committee of the Maseno University of Kisumu, Kenya (MSU/DRPC/MUERC/00054/13).

### Study Design and Participants

This study used data and biological specimens from a prospective cohort study of community-recruited heterosexual couples in Kisumu, Kenya. The details of recruitment have been previously described (Mehta et al., 2018). Briefly, cisgender male (aged 18–35 years) and female (aged 16 years and older) couples who had been in a sexual relationship for at least 6 months were recruited. The study was powered to estimate the effect of the penile microbiome on female sex partners’ risk of BV, with parameters of sample size estimation detailed in (Mehta et al., 2018).

Trained clinicians and counselors obtained standardized medical history, physical examination, and personal interviews to obtain information on sociodemographics and sexual behavior. Laboratory methods have been described in detail previously. Briefly, serum was tested for HSV-2 antibody (Kalon HSV-2 IgG ELISA, Kalon Biological Limited, Aldershot, UK) using the manufacturer’s recommended cutoff, a clinician-collected vaginal swab was used to assess BV according to Nugent’s criteria (Nugent et al., 1991), and testing for HIV infection was conducted using a serial rapid test protocol that followed the Kenyan national guidelines (National and Control Programme, 2015).

### Penile and Vaginal Microbiome Characterization

Clinicians twirled pre-moistened mini-tip flocked swabs (Copan Diagnostics, Inc., Corona, CA, USA) at the meatal opening for 3–5 rotations (Mehta et al., 2020). In uncircumcised men, the foreskin was retracted prior to sampling. The VMB was characterized in cervicovaginal lavage samples. Penile swabs and cervicovaginal lavage specimens were stored at −80°C until shipment. PCR amplification of the V3–V4 variable region of bacterial 16S rRNA genes was performed using a two-step PCR protocol with primers 341F and 806R, as described previously (Naqib et al., 2018). Amplicons were sequenced on an Illumina MiSeq instrument, implementing V3 chemistry (600 cycles). Forward and reverse reads were merged using the software package PEAR (Zhang et al., 2014). After standard read processing (i.e., quality trimming, primer removal, and chimera removal), annotation was conducted by the University of Maryland Institute for Genomic Science (Holm et al., 2019), as performed previously (Mehta et al., 2020). Subsequently, a biological observation matrix was generated at the lowest taxonomic level identifiable. Taxa identified as contaminants in negative controls [*via decontam*, (1.16.0) (Davis et al., 2018) in R] were removed, and samples with <5,000 reads (n = 19) were excluded. Data were filtered to retain taxa that contributed at least 0.1% of the total sequence reads, resulting in the retention of 54 penile taxa (from 2,138 taxa in the initial observation matrix). Raw sequence data files are available in the Sequence Read Archive (National Center for Biotechnology Information; BioProject identifier PRJNA 516684). Vaginal community state types (CSTs) were identified in a reference dataset using the nearest centroid classifier approach, VALENCIA (*VAginaL community state typE Nearest CentroId clAssifier*) (France et al., 2020). Of 252 men enrolled in the study, 249 men had microbiome data available. Participants with one visit were excluded from this analysis, resulting in 218 men contributing 740 observations: 47 with 2 visits, 58 with 3 visits, and 118 with 4 visits.

### Statistical Analysis

#### Inferential Analyses

Explanatory variables that were *a priori* hypothesized to be associated with the penile microbiome composition and change over time were tested: circumcision status, HIV status, HSV-2 status, condom use, number of sex partners, days since last sex, age, socioeconomic status (employment, educational attainment), female sex partner BV status, vaginal CST, and HIV and HSV-2 status.

First, we used permutational ANOVA (PERMANOVA) to test for differences in penile bacterial community composition between study time points using pairwise comparisons with correction for multiple testing (*RVAideMemoire* (0.9-81-2) (Herve, [[NoYear]]), in R) of the Bray–Curtis resemblance matrix. Next, hierarchical clustering was conducted to identify penile microbiome community types (CTs). Following methods recommended for reliable clustering (Koren et al., 2013), partition was applied around the medoid using five distance measures (Euclidean, Bray–Curtis, Mahalanobis, Jaccard, and Kulczynski). The optimum number of clusters that maximized both silhouette index (silhouette function (Desgraupes, 2017) in *cluster* (2.1.2) R package (Maechler et al., 2022), a measure of similarity of samples within their own cluster compared to other clusters) and prediction strength (*prediction.strength fpc* (2.2-9) (Hennig, [[NoYear]]), a validation test to check if the clusters are replicable in training and test sets) was selected. The optimal number of clusters based on silhouette index and prediction score was 7, using the Euclidean distance of the natural log-transformed RAs. Multinomial regression to identify changes in each of the 7 penile CTs over time and associated factors were used, with a random effect for subject intercept. Third, a linear mixed-effects model was used to test whether the RA (outcome) of the twelve meatal taxa with the highest mean RA was impacted by time and other subject-specific characteristics. Following geometric Bayesian multiplicative prior imputation of zeros [*zCompositions* (1.3.4) (Palarea-Albaladejo and Martin-Fernandez, 2015)], separate models were fitted with each center log ratio-transformed taxon as an outcome with a random effect for individual, and covariates were treated as fixed effects [*lme4* (1.1-27.1)] (Bates et al., 2015). Step-down Akaike information criterion (AIC) (Posada and Buckley, 2004) guided the final model selection. With the use of this same linear mixed model approach, factors associated with alpha diversity metrics over time were examined. Data were rarefied to 6,000 sequences prior to estimation of alpha diversity measures (*vegan* (2.5-7) (Oksanen et al., 2022)). Measures of Shannon, Simpson, and Evenness were highly correlated (0.94–0.96), and therefore, Shannon diversity index and richness were modeled due to their lower correlation (0.48–0.73) with the other alpha diversity measures. In all models, interactions with circumcision status and time, and with BV and time, were tested as an *a priori* hypothesis for association with stability and change. Fourth, group-based trajectories (GBTs) were estimated with zero-inflated Poisson regression for the four taxa with the highest RA (*Corynebacterium*, *Streptococcus*, *Staphylococcus*, and *Anaerococcus*) (Nagin and Odgers, 2010; Jones and Nagin, 2013). These GBTs shed light on longitudinal trends, by delineating stable and changing trajectories of RA. The number of groups and polynomial type for each trajectory was selected based on minimized AIC, although if an outlier group (<5% of observations) was produced, the number of groups was reduced. The final model selection for outcomes of CT, individual taxa, and alpha diversity measures was guided by minimizing AIC (Posada and Buckley, 2004). Lastly, the within-subject (i.e., paired) Bray–Curtis similarity was estimated from baseline to 6 months and from 6 months to 12 months, and multinomial mixed-effects modeling was used to assess factors associated with the Bray–Curtis similarity over time. The Bray–Curtis similarity was categorized as follows: 1) “less stable” included observations in the lowest 15th percentile and corresponded to <41% of Bray–Curtis similarity in paired measurements, 2) “stable” included observations between the 15th and 85th percentiles and corresponded to 41% to <70% similarity, and 3) “more stable” included observations in the highest 15th percentile of similarity and corresponded to >70% similarity. Final model selection for outcomes of CT, individual taxa, alpha diversity measures, and Bray–Curtis similarity was guided by minimizing AIC (Posada and Buckley, 2004).

#### Visualizations

Stacked bar charts, coefficient plots, and GBT plots were generated in Stata/SE 17. Change in the RA of individual taxa and alpha diversity measures across time by circumcision status and female partner BV status were visualized using line plots in R and visualization of transition of subjects across clusters with Sankey diagrams (*ggalluvial* (0.12.3) (Brunson and Read, 2020) in *ggplot2* (3.3.6) (Wickham, 2016)). The relationship of global bacterial communities was visualized by study time point using metric multidimensional scaling of bootstrapped averages of centroids with 100 replicates per group (Primer-E, version 7, UK). A beta coefficient heatmap was created in R using *ggplot* function within *ggplot2* (Wickham, 2016).

## Results

At baseline, men had a median age of 26 years, 11.6% were living with HIV, 46.1% were HSV-2 seropositive, and 55.5% were circumcised (**Table 1**). Female sex partners have a median age of 22 years, 10.2% were living with HIV, 55.4% were HSV-2 seropositive, 20.2% were with BV, and the majority (89.2%) of CSTs were either diverse (CST-IV, 46.5%) or *Lactobacillus iners* dominant (CST-III, 43.7%).

**Table 1:** Distribution of characteristics by study visit, N = 740 observations.

Visit	Baseline, N = 218 n (%)	1 month, N = 197 n (%)	6 months, N = 169 n (%)	12 months, N = 156 n (%)
*Male characteristics*				
Median age in years (IQR)	26 (24–30)	26.5 (24–30)	27 (24–30)	27 (24–30.75)
Educational attainment				
Primary or less	92 (42.2)	85 (43.2)	76 (45.0)	74 (47.4)
Some secondary or more	126 (57.8)	112 (56.9)	93 (55.0)	82 (52.6)
Currently employed	173 (79.4)	150 (76.1)	124 (73.8)	107 (69.9)
Circumcised	121 (55.5)	115 (58.4)	98 (58.0)	86 (56.6)
HIV positive	25 (11.6)	22 (11.3)	21 (12.5)	19 (12.3)
HSV-2 positive	100 (46.1)	91 (46.4)	97 (57.4)	94 (60.6)
Genital ulcer disease, past 6 months (self-reported or clinician detected)	8 (3.8)	3 (1.5)	4 (2.4)	5 (3.3)
Median days since last sexual intercourse (IQR)	4 (3–8)	4 (3–8)	6.5 (3–8)	8 (3–9)
When washed penis after last sex				
<1 h	49 (22.5)	48 (24.4)	47 (28.7)	49 (32.7)
1 h or more	169 (77.5)	149 (75.6)	117 (71.3)	101 (67.3)
Condom used at last sexual intercourse	36 (16.5)	36 (18.4)	32 (19.1)	30 (19.6)
Condom frequency in the past 6 months				
Never	134 (61.5)	122 (61.9)	105 (62.5)	98 (64.1)
Sometimes	69 (31.6)	63 (32.0)	52 (31.0)	45 (29.4)
Always	15 (6.9)	12 (6.1)	11 (6.5)	10 (6.5)
Number of sex partners in the past 6 months				
One	161 (74.9)	149 (76.4)	136 (85.0)	112 (78.3)
Two or more	54 (25.1)	46 (23.6)	24 (15.0)	31 (21.7)
*Alpha diversity metrics*, median (IQR)				
Shannon diversity index	1.86 (1.36–2.17)	1.80 (1.43–2.22)	1.80 (1.44–2.19)	1.77 (1.34–2.16)
Simpson diversity	0.76 (0.65–0.84)	0.76 (0.65–0.84)	0.76 (0.66–0.83)	0.75 (0.59–0.83)
Evenness	0.60 (0.50–0.68)	0.60 (0.52–0.69)	0.60 (0.48–0.68)	0.58 (0.46–0.66)
Richness	23 (16–27)	22 (16.5–27.5)	23 (17–28.5)	22 (18–28)
*Community type* (CT)				
CT-1 mixed	48 (22.0)	51 (25.9)	53 (31.4)	56 (35.9)
CT-2 *Corynebacterium* dominated	42 (19.3)	37 (18.8)	21 (12.4)	26 (16.7)
CT-3 *Streptococcus* dominated	19 (8.7)	14 (7.1)	12 (7.1)	13 (8.3)
CT-4 *Sneathia sanguinegens* dominated	33 (15.4)	28 (14.2)	19 (11.2)	14 (9.0)
CT-5 *Finegoldia*/*Anaerococcus* dominated	39 (17.9)	35 (17.8)	32 (18.9)	24 (15.4)
CT-6 *Lactobacillus iners* dominated	15 (6.9)	17 (8.6)	18 (10.6)	9 (5.8)
CT-7 Clostridiales family XII dominated	22 (10.1)	15 (7.6)	14 (8.3)	14 (9.0)
*Female partner characteristics*				
Median age in years (IQR)	22 (20–25)	22.5 (20–25)	23 (20–25)	23 (20–25)
Bacterial vaginosis (Nugent score 7–10)	43 (20.2)	44 (22.5)	41 (27.0)	32 (26.5)
Documented treatment for BV	40 (93.0)	41 (93.0)	39 (95.1)	27 (84.4)
Vaginal discharge	67 (31.2)	53 (27.0)	41 (26.8)	33 (26.0)
HIV positive	22 (10.2)	19 (9.7)	21 (13.2)	19 (14.8)
HSV-2 positive	121 (55.5)	108 (54.8)	101 (63.1)	88 (68.2)
Genital ulcer disease, past 6 months (self-reported or clinician detected)	23 (10.7)	6 (3.1)	7 (4.6)	5 (3.9)
*Community state type*				
I: *Lactobacillus crispatus* dominated	18 (8.5)	22 (12.0)	14 (9.2)	17 (13.3)
II: *Lactobacillus jensenii* dominated	2 (0.9)	1 (0.5)	2 (1.3)	1 (0.8)
III: *Lactobacillus iners* dominated	93 (43.7)	80 (43.7)	62 (40.8)	47 (36.7)
IV: *Gardnerella vaginalis* dominated	99 (46.5)	79 (43.2)	71 (46.7)	61 (47.7)
V: *Lactobacillus gasseri* dominated	1 (0.5)	1 (0.5)	3 (2.0)	2 (1.6)
Pregnant (urine HCG positive)	23 (10.7)	24 (12.2)	26 (16.6)	11 (8.9)
Contraceptive use				
Implant	62 (28.4)	57 (28.9)	50 (31.5)	41 (32.3)
Injectable	40 (18.4)	35 (17.8)	27 (17.8)	28 (22.1)
Median (IQR) days since last menstrual period among non-pregnant women	25 (10.5–52)	17 (6–42)	19.5 (7.25–42.75)	19 (7–49.25)
Median (IQR) days since last menstrual period among non-pregnant women, not using hormonal contraceptives	20 (10–36)	15 (4–30)	15 (6–29)	13.5 (5.75–31.25)

IQR, interquartile range; HSV-2, herpes simplex virus type 2; HCG, human chorionic gonadotropin; BV, bacterial vaginosis.

### Penile Community Type

**Figure 1:**
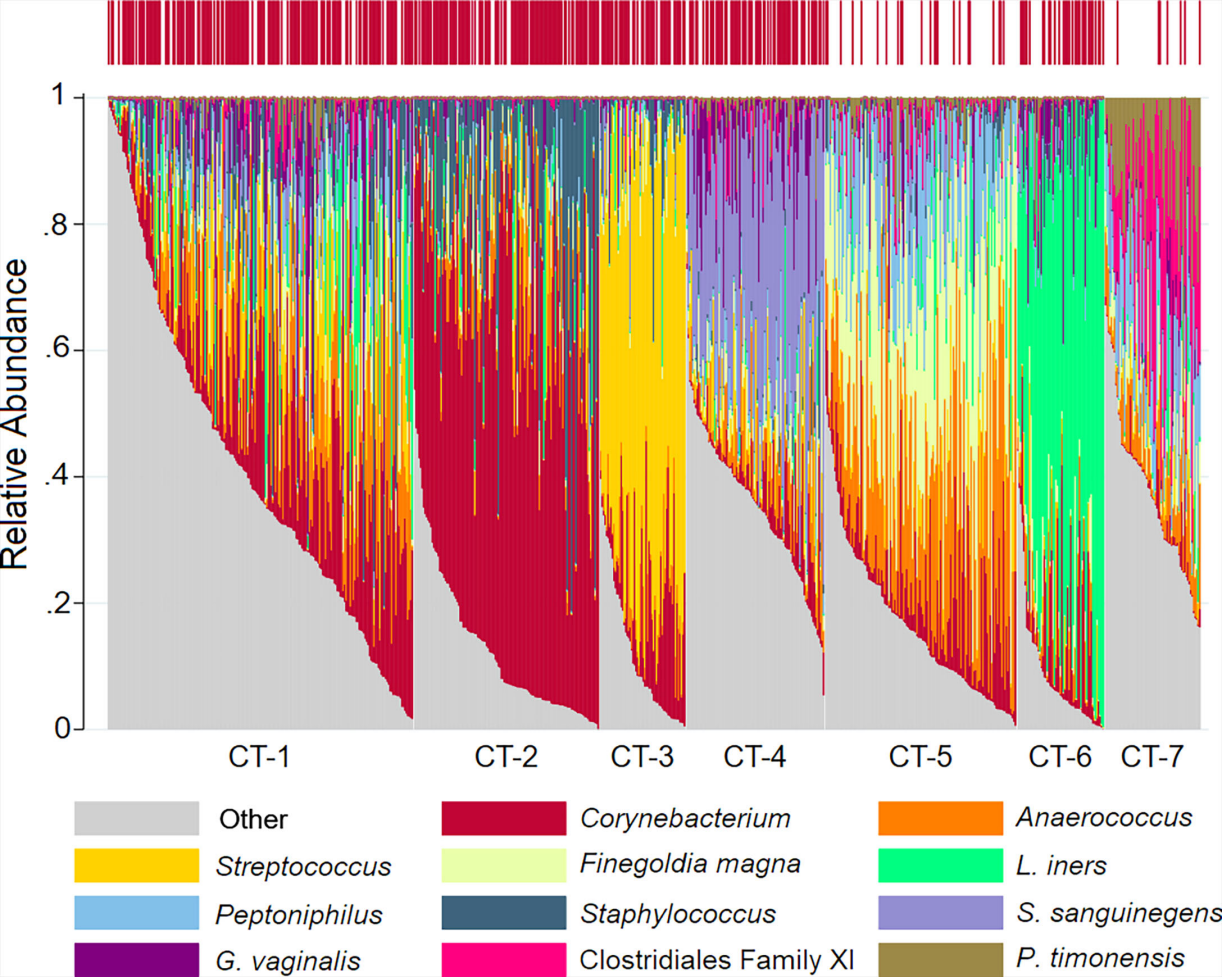
Stacked bar charts of the relative abundance of the penile taxa with greatest mean relative abundance for individuals by community type. Legend: observations from 740 samples are sorted by penile community type. The relative abundance of the taxa with the highest mean relative abundance is shown (0%–100%; y-axis), with individual subjects represented by individual bars, separated by community type (CT; x-axis). The bar at the top represents circumcision status, with circumcised individuals represented by red vertical lines.

We identified seven penile CTs (**Figure 1**, **Table 2**): mixed (CT-1), *Corynebacterium* dominated (CT-2), *Streptococcus* dominated (CT-3), *Sneathia sanguinegens* dominated (CT-4), *Finegoldia*/*Anaerococcus* dominated (CT-5), *L. iners* dominated (CT-6), and Clostridiales Family XI/*Prevotella timonensis* dominated (CT-7). Although clustering was unsupervised, clusters were delineated by circumcision status, emphasizing the role of circumcision as a driver of penile microbiome composition: just 7.7% and 4.6% of men in CT-5 and CT-7 were circumcised, respectively, compared to much higher proportions (60%–85.7%) in other CTs. CT-7 had the highest proportion of female partners with BV (36.4%) and with HIV (18.2%), the highest proportion of never using condoms (86.4%), and the lowest proportion of men with at least some secondary educational attainment (36.4%). By comparison, men in CT-1 had greater educational attainment (72.3% secondary or higher), the lowest prevalence of HSV-2 (35.4%), and a moderate prevalence of BV among female partners (21.2%). Compared to other CTs, for CT-2, CT-3, and CT-6, alpha diversity was decreased, BV prevalence among female partners was decreased, and the proportion of female partners with CST-I (*Lactobacillus crispatus* dominated) was increased.

**Table 2:** Distribution of baseline male and female partner characteristics by penile community type (CT).

	CT-1, N = 48	CT-2, N = 42	CT-3, N = 19	CT-4, N = 33	CT-5, N = 39	CT-6, N = 15	CT-7, N = 22
Taxa with highest mean relative abundance (mean RA, %)	*Corynebacterium* (15.2%) *Anaerococcus* (12.3%) *Streptococcus* (6.8%)	*Corynebacterium* (55.4%) *Staphylococcus* (15.8%)	*Streptococcus* (59.3%) *Corynebacterium* (9.1%)	*Sneathia sanguinegens* (32.9%) *Gardnerella vaginalis* (7.4%)	*Finegoldia magna* (27.8%) *Anaerococcus* (21.0%) *Peptoniphilus* (12.7%)	*Lactobacillus iners* (62.5%) *Corynebacterium* (11.1%)	Clostridiales Family XI (19.2%) *Peptoniphilus* (13.2%) *Prevotella timonensis* (11.1%)
Median years age (IQR)	26 (24–30)	27 (24–30)	26 (25–29)	25 (22–29)	27 (24–29)	27 (22–31)	26.5 (24–31)
Educational attainment							
Primary or less	13 (27.7)	24 (57.1)	6 (31.6)	16 (48.5)	17 (43.6)	7 (46.7)	14 (63.6)
Some secondary or more	34 (72.3)	18 (42.9)	13 (68.4)	17 (51.5)	22 (56.4)	8 (53.3)	8 (36.4)
Currently employed	37 (78.7)	32 (76.2)	15 (79.0)	29 (87.9)	32 (82.0)	10 (66.7)	18 (81.8)
Circumcised	34 (70.8)	36 (85.7)	14 (73.7)	24 (72.7)	3 (7.7)	9 (60.0)	1 (4.6)
HIV positive	3 (6.3)	7 (16.7)	1 (5.3)	3 (9.4)	6 (15.8)	3 (20.0)	2 (9.1)
HSV-2 positive	17 (35.4)	21 (50.0)	9 (47.4)	18 (56.3)	18 (46.2)	6 (40.0)	11 (50.0)
Condom frequency past 6 months^							
Never	30 (62.5)	20 (47.6)	10 (52.6)	19 (57.6)	25 (64.1)	11 (73.3)	19 (86.4)
Sometimes	14 (29.2)	17 (40.5)	7 (36.8)	13 (39.4)	12 (30.8)	3 (20.0)	3 (13.6)
Always	4 (8.3)	5 (11.9)	2 (10.5)	1 (3.0)	2 (5.1)	1 (6.7)	0 (0.0)
Condom used at last sex	9 (18.8)	12 (28.6)	3 (15.8)	2 (6.1)	8 (20.5)	2 (13.3)	0 (0.0)
Number of sex partners past 6 months							
One	40 (83.3)	35 (83.3)	12 (63.2)	18 (54.6)	30 (76.9)	12 (92.3)	14 (66.3)
Two or more	8 (16.7)	7 (16.7)	7 (36.8)	15 (45.4)	9 (23.1)	1 (7.7)	7 (33.3)
When washed penis after last sex							
<1 h	12 (25.0)	15 (35.7)	2 (10.5)	12 (36.4)	5 (12.8)	1 (6.7)	2 (9.1)
1 h or more	36 (75.0)	27 (64.3)	17 (89.5)	21 (63.6)	34 (87.2)	14 (93.3)	20 (90.9)
Median days since last sex prior to study visit (IQR)	3 (2–7.75)	5 (3–8)	5 (4–8)	4 (2–7)	4 (3–8)	4 (2–9)	5 (3–8.25)
Alpha diversity measures: Median (IQR)							
Shannon	2.05 (1.68–2.24)	1.35 (1.10–1.56)	1.33 (1.16–1.75)	2.08 (1.82–2.37)	2.01 (1.76–2.21)	1.24 (0.97–1.41)	2.09 (2.09–2.50)
Simpson	0.82 (0.74–0.85)	0.63 (0.49–0.68)	0.61 (0.46–0.74)	0.80 (0.76–0.86)	0.81 (0.75–0.83)	0.57 (0.49–0.67)	0.87 (0.80–0.89)
Evenness	0.66 (0.59–0.70)	0.49 (0.40–0.55)	0.52 (0.38–0.59)	0.63 (0.58–0.69)	0.61 (0.56–0.66)	0.44 (0.38–0.51)	0.72 (0.66–0.75)
Richness	22.5 (18–25.75)	16 (14–19.75)	16 (13–24)	27 (23–34)	26 (21–31)	17 (11–22)	26 (23.5–29)
*Female partner characteristics*							
Median age in years (IQR)	22 (20–26)	22 (20–26.25)	23 (21–24)	22 (20–25)	24 (20–25)	21 (20–22)	24 (20–25.25)
HIV positive	4 (8.5)	4 (9.5)	0 (0.0)	3 (9.4)	5 (12.5)	2 (13.3)	4 (18.2)
HSV-2 positive	24 (50.0)	21 (50.0)	9 (47.4)	24 (72.7)	20 (51.3)	11 (73.3)	12 (54.6)
Nugent Score							
0–3	35 (74.5)	29 (69.0)	11 (64.7)	19 (61.3)	28 (71.8)	7 (46.7)	12 (54.6)
4–6	2 (4.3)	7 (16.7)	4 (23.5)	3 (9.7)	5 (12.8)	6 (40.0)	2 (9.1)
7–10	10 (21.8)	6 (14.3)	2 (11.8)	9 (29.0)	6 (15.4)	2 (13.3)	8 (36.4)
Community State Type							
I: *Lactobacillus crispatus*	1 (2.1)	4 (9.5)	2 (11.8)	2 (6.2)	7 (18.0)	1 (6.7)	1 (4.8)
II: *Lactobacillus gasseri*	0 (0.0)	0 (0.0)	0 (0.0)	1 (3.1)	1 (2.6)	0 (0.0)	0 (0.0)
III: *L. iners*	23 (46.8)	23 (54.8)	8 (47.1)	10 (31.3)	14 (35.9)	8 (53.3)	8 (38.1)
IV: *G. vaginalis*	24 (51.1)	14 (33.3)	7 (41.2)	19 (59.4)	17 (43.6)	6 (40.0)	12 (57.1)
V: *Lactobacillus jensenii*	0 (0.0)	1 (2.4)	0 (0.0)	0 (0.0)	0 (0.0)	0 (0.0)	0 (0.0)
Pregnant (urine HCG positive)	2 (4.2)	5 (11.9)	1 (5.3)	3 (9.4)	2 (5.4)	3 (20.0)	7 (31.8)
Contraceptive use							
Implant	12 (25.0)	11 (26.2)	4 (21.1)	10 (30.3)	14 (35.9)	6 (40.0)	5 (22.7)
Injectable	11 (22.9)	8 (19.1)	0 (0.0)	6 (18.2)	8 (20.5)	2 (13.3)	5 (22.7)
Median (IQR) days since last menstrual period among non-pregnant women	24 (10–55.75)	23.5 (8.25–112)	15 (6–23.75)	18 (6.5–31.5)	14.5 (6.25–39.5)	21 (8.25–40.75)	17 (4–35)
Median (IQR) days since last menstrual period among non-pregnant women, not using hormonal contraceptives	15 (5–42)	18 (8–38)	16.5 (5.75–21.5)	19 (5–25.75)	17 (7–52)	16 (9–25)	6.5 (3–18.5)

IQR, interquartile range; HSV-2, herpes simplex virus type 2.

^Because so few men reported “always” using condoms, “sometimes” and “always” were combined in analyses.

### Factors Associated With Penile Community Type Over Time

**Figure 2:**
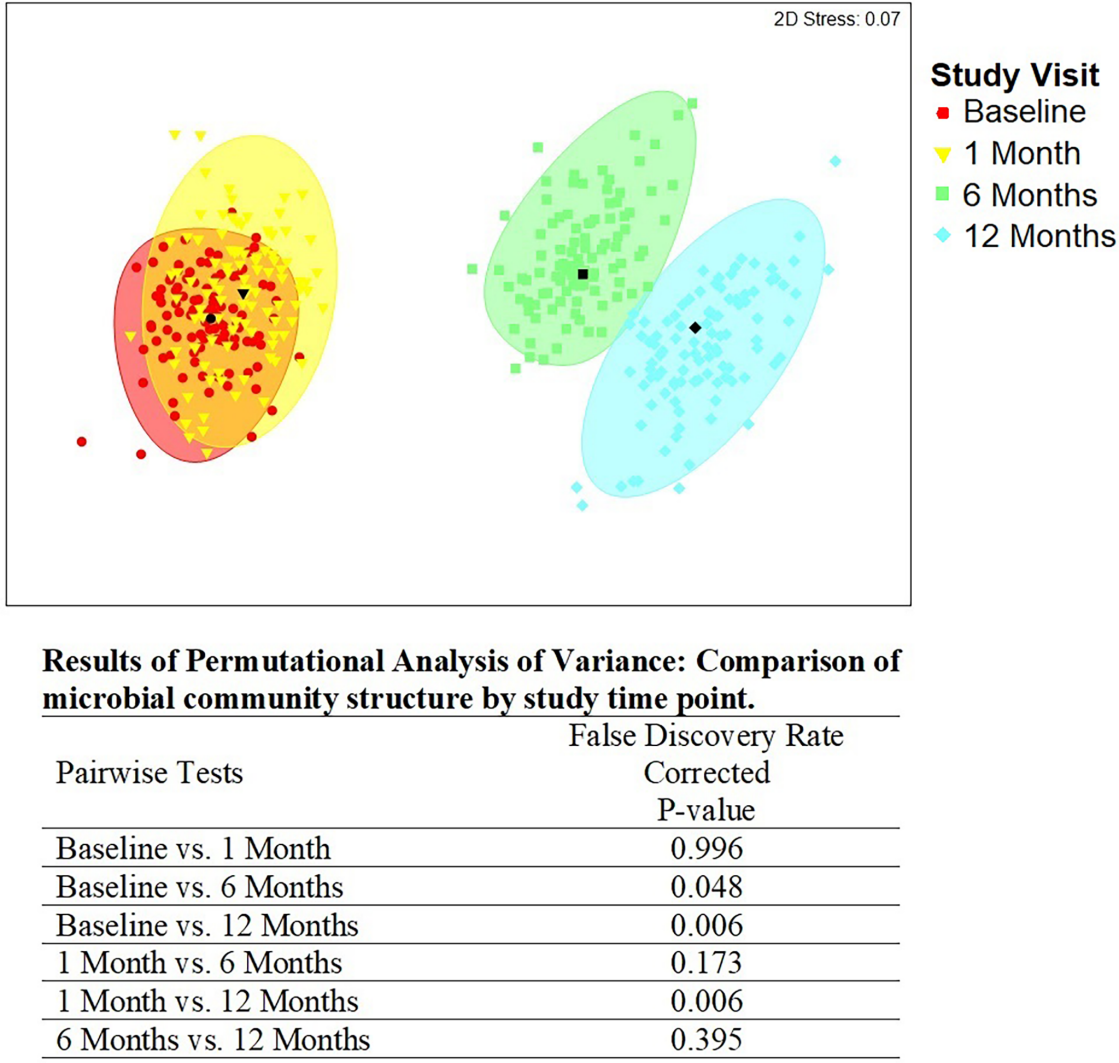
Metric multidimensional scaling plot of penile meatal microbial community by study time point. Legend: the four different colors represent the four study time points: red = baseline; yellow = 1 month; green = 6 months; blue = 12 months. Each colored mark indicates one of 100 bootstraps of the dataset. The matching shaded area represents 95% coverage. The black symbol at the center of each colored shape represents the average centroid of the 100 bootstraps. The false discovery rate-corrected p-values of the pairwise comparisons by time point are shown below the figure.

**Figure 3:**
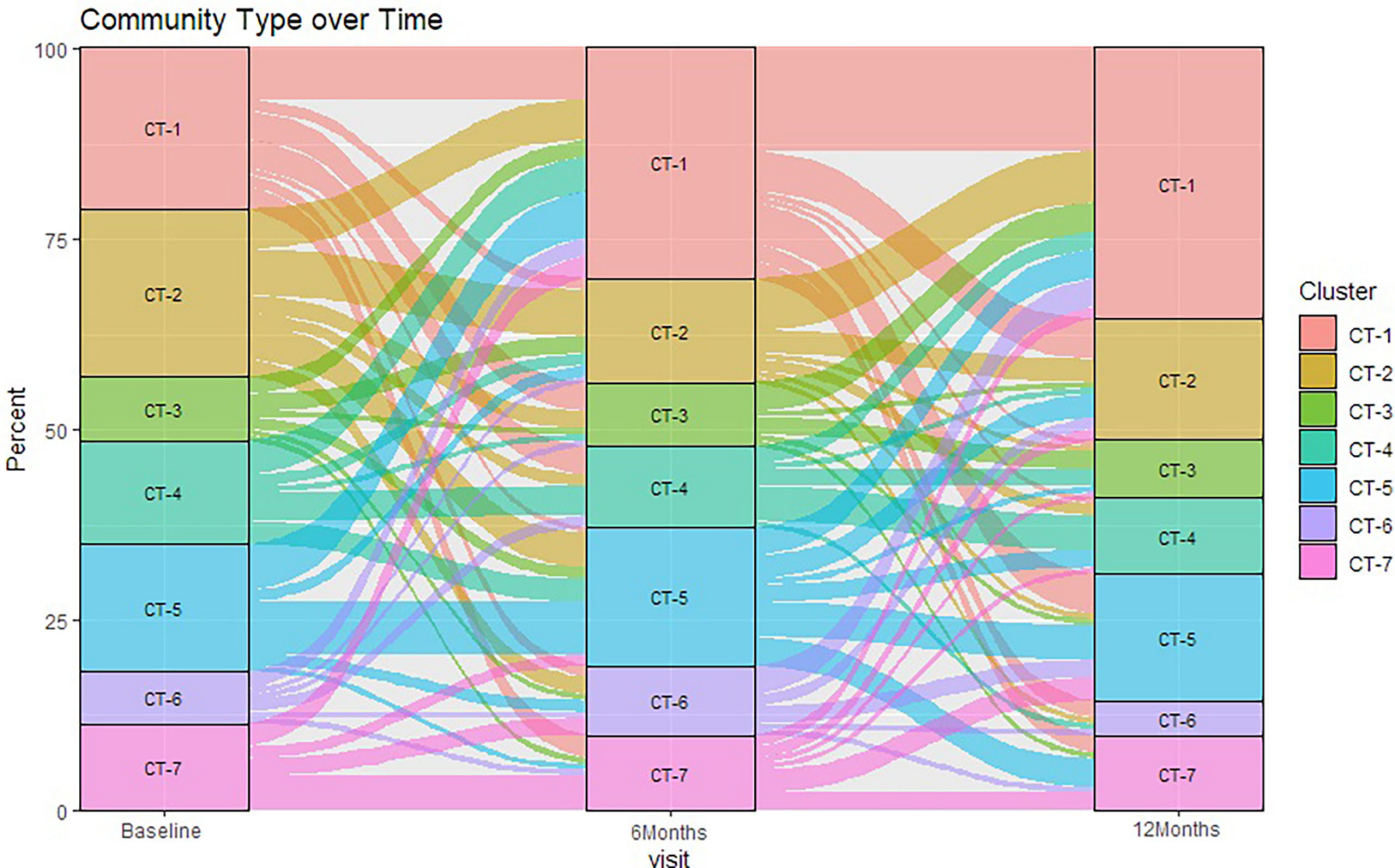
Sankey diagram showing change in penile meatal community type (CT) over time. Legend: the percent of samples (y-axis) classified in seven penile meatal community types (CT) over time (x-axis) are represented by seven colored blocks, with key to the right.

There were statistically significant (p < 0.05) changes over time from baseline and 1-month visits to 6- and 12-month visits (**Figure 2**). Additionally, alluvial visualization indicates that individuals changed meatal CT over time (**Figure 3**), with an increasing proportion of individuals in CT-1. In multivariable-adjusted analyses (**Table 3**), relative to CT-2 (*Corynebacterium* dominated), CT-1 (mixed) increased over time relative to baseline, with transient increases in CT-5 (*Finegoldia* dominated) and CT-6 (*L. iners* dominated) at 6 months. Relative to CT-2, men were less likely to be circumcised if they were in CT-4 (*S. sanguinegens* dominated; adjusted odds ratio (aOR) = 0.45), CT-5 (aOR = 0.04), CT-6 (aOR = 0.28), and CT-7 (Clostridiales Family XI/*P. timonensis*; aOR = 0.03). Men who reported using condoms at the last sex act were less likely to have penile CT-4 than CT-2 (aOR = 0.43). Men who reported washing their penis more than 1 h after sex were more likely to have CT-5 (aOR = 2.39), CT-6 (aOR = 2.61), or CT-7 (aOR = 2.31) penile microbiome than CT-2. The association of vaginal CST-IV with penile CT-7 (aOR = 5.30) remained statistically significant in adjusted analyses. Univariate results were largely similar in terms of the magnitude of associations and significance (**Supplementary Table 1**).

**Table 3:** Results of multivariable mixed effects multinomial modeling*: adjusted^ odd ratio and 95% CI of penile meatal community type.

	CT-1 Mixed vs. CT-2 aOR (95% CI)	CT-3 *Streptococcus* dominated vs. CT-2 aOR (95% CI)	CT-4 *Sneathia sanguinegens* dominated vs. CT-2 aOR (95% CI)	CT-5 *Finegoldia*/*Anaerococcus* dominated vs. CT-2 aOR (95% CI)	CT-6 *Lactobacillus iners* dominated vs. CT-2 aOR (95% CI)	CT-7 Clostridiales/*Peptoniphilus* dominated vs. CT-2 aOR (95% CI)
Circumcised (vs. uncircumcised)	0.57 (0.29–1.13)	0.53 (0.22–1.27)	**0.45** (0.22–0.96)^b^	**0.04** (0.02–0.09)^a^	**0.28** (0.12–0.65)^a^	**0.03** (0.01–0.07)^a^
Condom used at last sexual act	0.69 (0.35–1.39)	0.69 (0.26–1.80)	**0.43** (0.18–1.02)^c^	1.04 (0.47–2.31)	0.65 (0.25–1.71)	0.74 (0.28–1.98)
Washed penis more than 1 h after last sex (vs. ≤1 h)	1.32 (0.71–2.44)	1.26 (0.55–2.89)	0.88 (0.45–1.75)	**2.39** (1.10–5.20)^b^	**2.61** (1.03–6.59)^b^	**2.31** (0.94–5.63)^c^
Female partner CST (vs. CST-I)						
CST-III	1.87 (0.73–4.77)	0.62 (0.21–1.86)	1.55 (0.52–4.67)	1.00 (0.36–2.73)	1.27 (0.41–3.93)	3.81 (0.70–20.6)
CST-I–IV	2.05 (0.80–5.24)	0.65 (0.22–1.95)	1.85 (0.62–5.56)	0.82 (0.30–2.25)	0.75 (0.23–2.41)	5.30 (1.00–28.1)^b^
Study visit (vs. baseline)						
1 month	1.36 (0.70–2.61)	0.89 (0.36–2.20)	1.21 (0.58–2.52)	1.15 (0.54–2.47)	1.32 (0.54–3.23)	0.92 (0.37–2.33)
6 months	2.40 (1.14–5.05)^a^	1.47 (0.54–4.01)	1.39 (0.59–3.28)	2.21 (0.94–5.21)^c^	2.62 (0.99–6.89)^c^	1.91 (0.70–5.18)
12 months	2.05 (0.98–4.29)^c^	0.94 (0.33–2.68)	0.93 (0.38–2.26)	0.97 (0.39–2.42)	1.07 (0.36–3.21)	1.43 (0.52–3.88)

Final adjusted sample consists of 654 observations among 219 subjects.

aOR, adjusted odds ratio.

^The model is simultaneously adjusted for all variables presented.

*Reference category is community type 2 (Corynebacterium dominated).

a p < 0.01.

b p < 0.05.

c 0.05 < p < 0.10.

### Change in Individual Penile Taxa Over Time and Associated Factors

**Figure 4:**
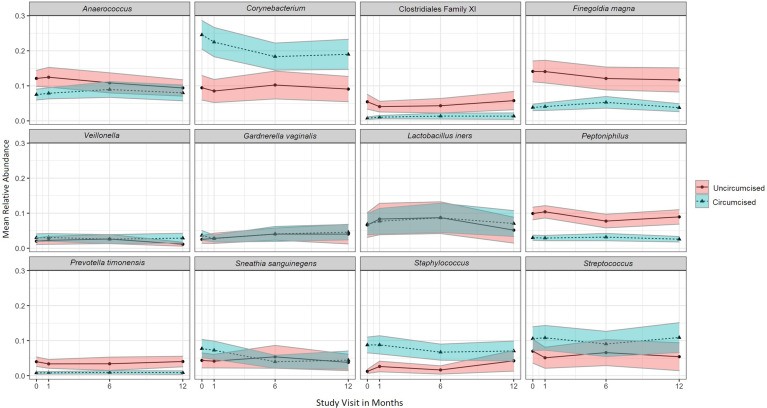
Mean relative abundance over time for the twelve penile meatal taxa with highest mean relative abundance, by circumcision status. Legend: the mean relative abundance of the twelve penile meatal taxa with highest mean relative abundance (RA; y-axis) are shown over time (x-axis) with the 95% CI shown in the shaded areas around the mean line. Mean relative abundances over time are stratified by circumcision status, as represented by legend to the right of the graphs.

**Figure 5:**
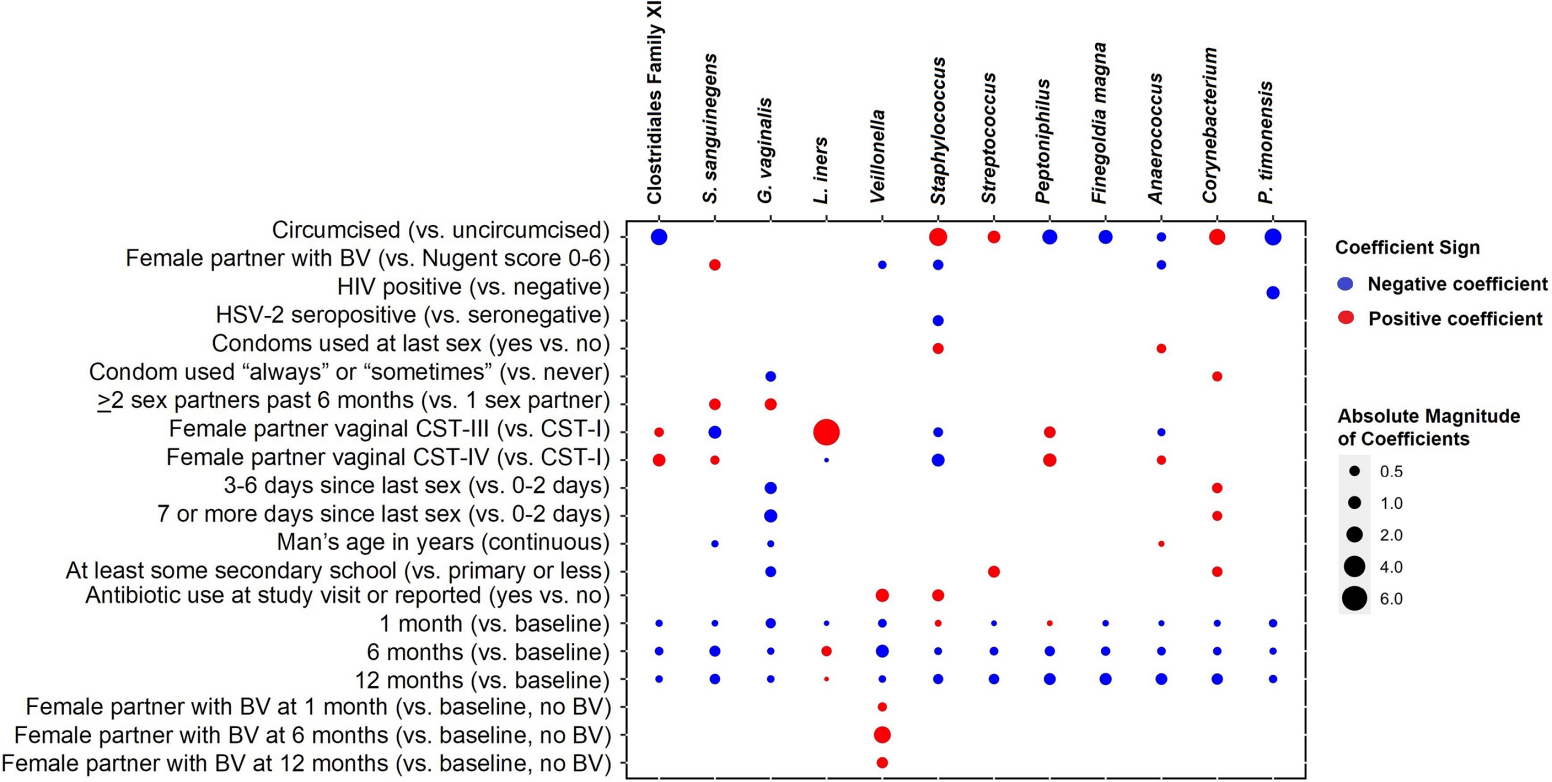
Coefficient heatmap showing results of multivariable-adjusted random-effects regression: factors associated with 12 taxa with highest mean relative abundance over time. Legend: the columns represent the 12 taxa with the highest mean relative abundance. The rows represent the covariates associated with taxa in multivariable models that are time-adjusted, with final models selected according to minimized Akaike’s information criterion. A red circle represents a positive association between covariate and taxon, and a blue circle represents a negative (inverse) association. The size of the dot is proportional to the absolute magnitude of the coefficient. White space indicates that the association between covariate and taxon did not improve explanation and is not included in the final model.

The twelve taxa with the highest mean RA accounted for 77.0% of the overall mean RA, with *Corynebacterium* (16.2%), *Anaerococcus* (9.4%), *Streptococcus* (8.5%), *Finegoldia* (8.0%), and *L. iners* (7.4%) having the greatest mean RA (**Figure 4**). Results of multivariable models are summarized visually (**Figure 5**; **Supplementary Table 2** for tabular details). On average, changes over time were not statistically significant for most taxa; only the RAs of *Corynebacterium* and *Anaerococcus* changed, both declining over time. Men who were circumcised had higher RA of *Corynebacterium*, *Streptococcus*, and *Staphylococcus* and lower RAs of several anaerobic bacteria. Unsurprisingly, CST-III (*L. iners* dominant) in female sex partners had a strong positive association with the RA of penile *L. iners*. Compared to men whose sex partners had CST-I (*L. crispatus* dominant), those whose partners had CST-III and CST-IV (*Gardnerella vaginalis* dominant) had decreased RA of *Staphylococcus* and increased RA of anaerobes.

### Trajectories of Taxa Over Time and Associated Factors

**Figure 6:**
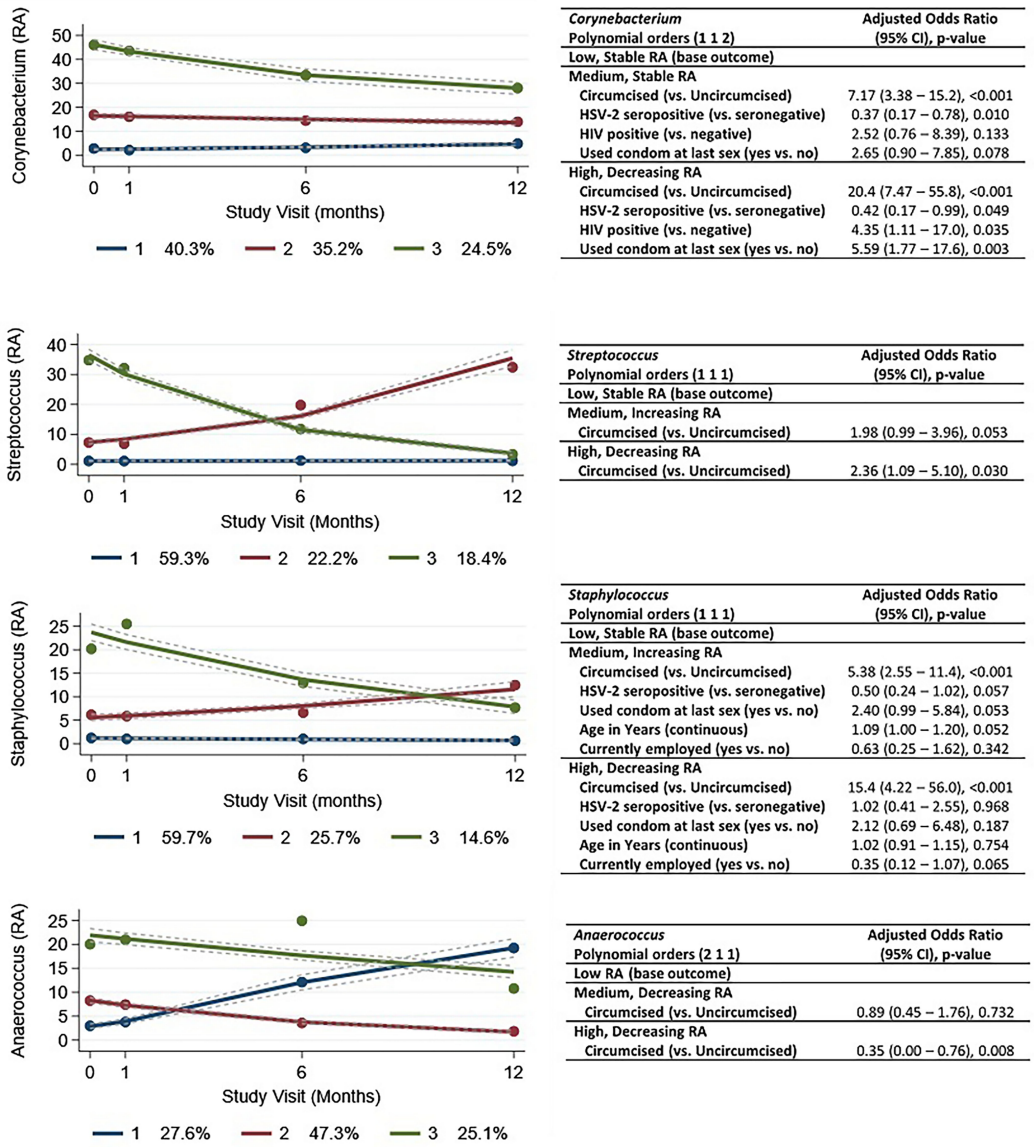
Group-based trajectories and results of multivariable multinomial modeling of factors associated with trajectory. Legend: the figures show the estimated group trajectories of relative abundances of the four taxa with highest relative abundance: **(A)** *Corynebacterium*, **(B)** *Streptococcus*, **(C)** *Staphylococcus*, and **(D)** *Anaerococcus*. The y-axis represents relative abundance (RA), and the x-axis represents time in months, with study visits indicated. Each trajectory is represented by a different color (blue, red, or green) with group membership shown in the legend below each figure. For example, for *Corynebacterium*, there is a stable trajectory with low relative abundance (dark blue), and 40.3% of observations are grouped in this trajectory. There is a stable, “medium” relative abundance (dark red), and 35.2% of observations are grouped in this trajectory. There is a (dark green) “high” relative abundance trajectory (with decline over time), and 24.5% of observations are grouped in this trajectory. The tables to the right of each figure (i–iv) show the results of multivariable multinomial modeling of baseline factors associated with group trajectories. For *Corynebacterium*, compared to men who are grouped in the stable, low RA group trajectory, men who are grouped in the stable, “medium” RA trajectory are more likely to be circumcised (adjusted odds ratio 7.17) and less likely to be HSV-2 seropositive (adjusted odds ratio = 0.37). Men who are grouped in the decreasing, “high” RA trajectory are more likely to be circumcised (adjusted odds ratio 20.4), less likely to be HSV-2 seropositive, more likely to be HIV positive, and more likely to report condom use at the last sexual encounter.

We identified three trajectories for each of the four taxa with the highest mean RA (**Figure 6**). Within *Corynebacterium*, 40.3% of men exhibited a stable, low RA trajectory and 35.2% with a stable, medium RA trajectory. Approximately a quarter of men (24.5%) were classified into a high RA trajectory that decreased over time. *Staphylococcus* and *Streptococcus* trajectories were similar, with a majority of men (59.7% and 59.3%, respectively) having a stable, low RA trajectory; the proportions of men with medium and high RA trajectories were also similar, which is in keeping with the correlation between *Staphylococcus* and *Streptococcus* RA (**Supplementary Figure 1**). Although *Anaerococcus* also had “low,” “medium,” and “high” RA trajectory groups, the low RA trajectory (27.6% of men) showed substantially increasing RA over time, and the medium RA (47.3%) and high RA (25.1% of men) groups showed decreasing RA over time. In general, our models appear to identify factors associated with composition rather than changing trajectory. For example, men grouped into the high, decreasing RA trajectory of *Corynebacterium* were more likely to be circumcised (aOR = 20.4) and less likely to be HSV-2 seropositive (aOR = 0.42), which are two factors with few time-varying values. Aside from circumcision status, models failed to identify factors associated with *Staphylococcus* and *Anaerococcus* trajectories.

### Change in Alpha Diversity Measures Over Time and Associated Factors

**Figure 7:**
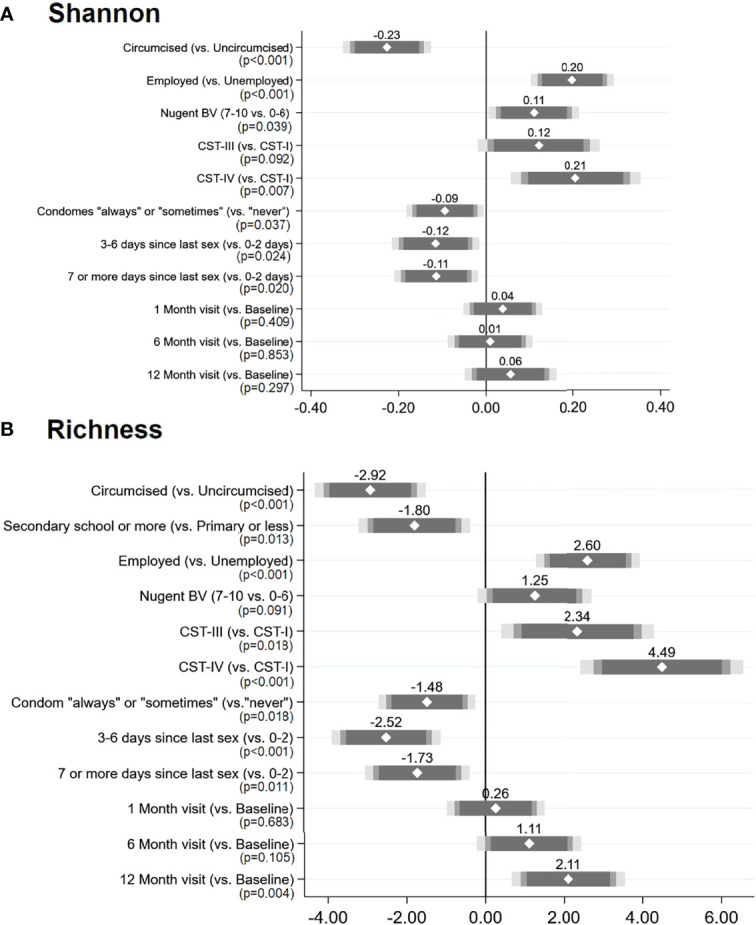
Results of multivariable adjusted mixed-effects linear regression: factors associated with penile meatal microbiome alpha diversity measures over time: **(A)** Shannon diversity index and **(B)** richness. Legend: the coefficient plots reflect the coefficient (white diamond) placed over the 95% CI (gray-shaded bars) for each factor (y-axis) in relation to the alpha diversity measures: **(A)** Shannon diversity index and **(B)** richness. p-Values are indicated under each variable. N = 654 observations among 221 individuals in both models.

In univariate analysis, alpha diversity measures did not statistically significantly vary over time (**Supplementary Figure 2**, **Supplementary Table 3**). In adjusted mixed-effects modeling (**Figure 7**, **Supplementary Table 3**), variables showed similar patterns of association with Shannon diversity and richness. Circumcised men had decreased alpha diversity measures, as did those who reported condom use, and a longer time since last sexual intercourse. Employed men and those whose female partners had BV or a VMB classified as CST-III or CST-IV (vs. CST-I) had increased alpha diversity measures. Shannon diversity index did not change over time, and there were no statistically significant or meaningful interactions of circumcision status or female partner BV with time. A statistically significant increase in richness (coefficient = 1.96) was observed at the 12-month visit in the adjusted model: this was due to an interaction over time with the female partner’s BV status. Although overall penile microbiome richness was greater for men whose partners had BV, there was a reduction in penile richness at the 12-month visit for men whose partners had BV and an increase in richness for men whose partners did not have BV; a similar but non-significant trend was observed for Shannon diversity index (**Supplementary Figure 3**).

### Paired Measures of Bray–Curtis Similarity Over Time

The mean Bray–Curtis similarity was 56.8% for paired observations between 0 and 6 months and 56.3% between 6 and 12 months. In multivariable mixed-effects regression (**Supplementary Table 4**), men with less stable (<41%) Bray–Curtis similarity were more likely to have HSV-2 (aOR = 2.99) and less likely to be currently employed (aOR = 0.42). Only two variables were marginally associated with higher stability (>71%): increasing age was inversely associated (aOR = 0.92; 95% CI: 0.84–1.01), as was more frequent condom use (aOR = 0.50; 95% CI: 0.23–1.11). Circumcision status, female partner BV status and VMB CST, HIV status (male or female), and sexual practices were not associated with the Bray–Curtis similarity between visits.

## Discussion

This study had two major findings, regarding 1) stability of the penile microbiome and 2) longitudinal factors shaping the penile microbiome composition. A substantial proportion of men had stable penile microbiome composition over 1 year as reflected by GBT estimation, individual taxa, and alpha diversity measures. In keeping with the GBT analyses, taxa with the highest mean RA were relatively stable over time for the study sample overall, except for modest decreases in *Corynebacterium* and *Anaerococcus*, concomitant with an increasing proportion of men with CT-1 and decreasing CT-2. Through a series of analyses, we identified that circumcision status, HSV-2, condom use, and female sex partner VMB composition are important factors influencing penile microbiome composition and alpha diversity over a 1-year period, which is the longest period of observation of the penile microbiome that we are aware of.

There can be substantial fluctuation in daily (Srinivasan et al., 2010; Song et al., 2020) and weekly (Srinivasan et al., 2010; Mirmonsef et al., 2015) VMB composition, although some studies have shown that community compositions can be quite stable over a period of months (Gajer et al., 2012). VMB composition is temporally influenced by the stage of the menstrual cycle, sexual activity, type of hormonal contraceptive use, diet, exercise, and antibiotic use. In our study, sexual practices (number of sex partners, condom use, and time since the last sex act) were associated with penile microbiome composition. This is intuitive, as these activities represent direct exposure to another microbiome. However, within this cohort of couples who had been sexually active together for at least 6 months (with a median relationship duration of 3 years as reported previously (Mehta et al., 2018)), there was little change in sexual practices (condom use and having multiple sex partners). Nelson et al. also observed a stable penile microbiome at monthly visits in their study of 18 male participants aged 14–17 years, with 61% sexually active (Nelson et al., 2012). Unlike the VMB, the penile microbiome may have fewer factors regulating homeostasis or may be less susceptible to perturbation in general.

Antibiotic use was not consistently associated with penile microbiome composition in our cohort (**Supplementary Tables** and **Figure 5**), although only a small number of men were given and/or reported taking antibiotics. Because of the evidence for the sexual exchangeability of the penile microbiome and VMB and their relation to BV, studies are examining the effects of antibiotics on the penile microbiome. Whether oral or topical antibiotic regimens similar to those used by women to treat BV can affect change in the penile microbiome composition persisting for 4 weeks was evaluated by Galiwango et al. among 125 HIV-negative Ugandan men (Galiwango et al., 2019). In a pilot study of 34 heterosexual couples followed up for 12 weeks, Plummer et al. evaluated the effect of standard antimicrobial treatments for BV in women (oral metronidazole and clindamycin cream) on the penile microbiome of men whose partners were treated for BV (Plummer et al., 2021). The presence and abundance of BV-associated bacteria were reduced, and *Staphylococcus* increased in men’s first-pass urine (representing the urethra), but this was not sustained by 12 weeks posttreatment, despite 81% of women having suppression of BV-associated bacteria over the 12-week period. These findings highlight the need to characterize the male genitourinary microbiome over time and associated factors to understand why effects may not be sustained and to better select and design therapeutic or interventional options related to microbiome manipulation. As evidence supports the sexual exchangeability of the penile microbiome and VMB, antibiotic treatment to modify the penile microbiome may need to be simultaneously administered to female partners. In our study, since 92% of instances of BV in female partners were treated with antibiotics, we were unable to assess the impact of female partner treatment for BV on the penile microbiome. However, this may explain the decline in alpha diversity among men whose partners had BV (**Supplementary Figure 3**).

Although standard penile CTs have not been established in the same way as vaginal CSTs, our findings share some similarities to dominant penile CTs identified in two cross-sectional studies. Among Ugandan men (Liu et al., 2017), 7 CTs were identified: *Corynebacterium* and *Staphylococcus* were more prevalent and abundant in CT-1 to CT-3, while men with the other four CTs had higher prevalence and abundance of *Porphyromonas*, *Prevotella*, and Clostridiales Family XI. Men with penile CTs with greater presence and abundance of anaerobes were more likely to have multiple sex partners and female partners with BV. Among South African men (Onywera et al., 2020a), 6 CTs were identified: the most common CT was *Corynebacterium* dominated, present in 53.4% of men. Other CTs were *Lactobacillus* dominated (2.5% of men), *Prevotella* dominated (18.5% of men), *Gardnerella* dominated (8.8% of men), mixed (9.2% of men), and *Chryseobacterium* dominated (7.6% of men). As in our study, alpha diversity was significantly lower for the *Corynebacterium*-dominated CT, and BV-associated bacteria were more predominant in non-*Corynebacterium*-dominated communities. Men with *Corynebacterium*-dominated CT were also less likely to harbor high-risk HPV. Differences across studies in penile CT identification and definition may arise from how the penis is sampled; the DNA extraction, sequencing, and annotation processes; and the statistical approach to clustering. For comparability across studies, we need a standardized definition of penile community type, similar to that which has emerged for the VMB (McKinnon et al., 2019; France et al., 2020). Nevertheless, our study and others indicate that a *Corynebacterium*-dominated, low-diversity penile microbiome community may have beneficial health associations for men and their female partners.

In the female genital tract, a diverse VMB is often associated with non-optimal VMB and poorer health outcomes (McKinnon et al., 2019). Our results contribute toward understanding whether this framework extends to the penile microbiome and whether diversity and the associated penile microbiome compositions influence susceptibility or resilience to poorer health outcomes in men. Penile microbiome alpha diversity was increased when female partners had BV and non-optimal CST, and men with CT-4 and CT-7 (highest Shannon diversity) had the highest prevalence of HSV-2. We observed decreased alpha diversity with circumcision and condom use, indicating that these factors may contribute to the stability of the penile microbiome. Among South African men, alpha diversity was increased among men dually infected with HIV and human papillomavirus (Onywera et al., 2020a). While more research is needed, these results suggest that greater diversity of the penile microbiome may be part of a non-optimal penile microbiome composition. Understanding what constitutes an optimal or non-optimal penile microbiome and identifying intrinsic (e.g., host interactions) and extrinsic (e.g., behavioral and environmental) mediating or modifying factors as has been done for the VMB (Murphy and Mitchell, 2016; McKinnon et al., 2019) will require longitudinal studies defining the penile microbiome and effects on men’s health. Building this knowledge base will have important implications for the health of men and their female partners.

### Strengths and Limitations

As reviewed in (Onywera et al., 2020b), most studies of the penile microbiome have been cross-sectional or longitudinal with a small sample size and/or limited duration. In this large, well-characterized longitudinal cohort of community-recruited young Kenyan men and their female partners, we conducted repeated penile microbiota assessments and multifaceted statistical analyses. Although we were able to measure change over time, it was not possible to measure the penile VMB and VMB prior to sexual activity and determine conclusive directionality; even if we had asked couples to refrain from sexual intercourse for a period of time prior to sampling, and then obtain penile and vaginal samples within a short period of time after intercourse, this would not be a true “baseline” because couples were already exposed to each other’s microbiota. Moreover, such a study would have been unethical in this setting, as couples may feel coerced into unwanted sexual practices. This study had limitations inherent to microbiome studies, including measurement error in the assignment of taxa. Unmeasured behavioral, dietary, immunologic, genetic, and other health-related factors that may shape the penile microbiome or could confound the associations observed need to be studied.

## Conclusions

The penile microbiome was stable over 1 year for at least half of men; and stable or varying composition was influenced by circumcision, HSV-2, female partner BV status and vaginal CST, and sexual practices. Identification of an optimal penile microbiome through association with health outcomes, a meta-transcriptome study to understand the activity of key taxa, and a comprehensive measure of factors shaping the penile microbiome are necessary to consider interventions to prevent adverse outcomes related to the non-optimal penile microbiome in men and associated conditions in female sex partners.

## Data Availability Statement

The datasets presented in this study can be found in online repositories. The names of the repository/repositories and accession number(s) can be found in the article/**Supplementary Material**.

## Ethics Statement

This study was approved by the Institutional Review Board of University of Chicago, Illinois USA (2013-0511) and Ethical Review Committee of Maseno University of Kisumu, Kenya (MSU/DRPC/MUERC/00054/13). Written informed consent from the participants’ legal guardian/next of kin was not required to participate in this study in accordance with the national legislation and the institutional requirements.

## Author Contributions

SM: obtained funding, study conceptualization and design, implementation of statistical analysis approaches (mixed-effects modeling of alpha diversity, Bray–Curtis similarity), visualization (stacked bar chart, non-metric multidimensional scaling (NMDS), and alluvial diagram), and drafting of the manuscript. DN: design and implementation of statistical analysis approach (specifically hierarchical clustering, mixed-effects modeling of individual taxa, and GBTs), visualization (correlation matrix, coefficient heatmap, taxa, and alpha diversity over time), and critical review and revision of the manuscript. SG: development and oversight of protocols for amplicon sequencing, microbiologic analyses and interpretation, and critical review and revision of the manuscript. WA: development, implementation, and oversight of laboratory protocols in Kenya; acquisition of data; microbiologic analyses and interpretation; and critical review and revision of the manuscript. FO: study oversight and management to ensure integrity to protocols and critical review and revision of the manuscript. DB: conceptualization and design of statistical analysis approach (specifically evaluating overall approach and results) and critical review and revision of the manuscript. RB: study oversight and management to ensure integrity to protocols and critical review and revision of the manuscript. All authors contributed to the article and approved the submitted version.

## Funding

This study was supported by grant number R01-AI110369 (PI: Mehta) from the National Institutes of Health, National Institute of Allergy and Infectious Diseases, Division of Microbiology.

## Conflict of Interest

The authors declare that the research was conducted in the absence of any commercial or financial relationships that could be construed as a potential conflict of interest.

## Publisher’s Note

All claims expressed in this article are solely those of the authors and do not necessarily represent those of their affiliated organizations, or those of the publisher, the editors and the reviewers. Any product that may be evaluated in this article, or claim that may be made by its manufacturer, is not guaranteed or endorsed by the publisher.

## Supplementary Material

Supplementary Figure 1 Correlation heatmap showing the Spearman correlations among twelve taxa with highest relative abundance and selected covariates of importance. Spearman rank correlation heatmap of the top 12 penile taxa with greatest relative abundance, and selected covariates. Relative abundance is center log ratio transformed prior to correlation estimation. The correlation heat map represents the direction and magnitude of the Spearman rank correlation between the taxa and covariates. Negative correlations are shaded in blue and positive correlations in red, with deeper intensity representing the magnitude of the correlation. Taxa are represented at the genus level, except where species is noted, for the following: *Prevotella timonensis, Sneathia sanguinegens, Gardnerella vaginalis, and Lactobacillus iners.*

Supplementary Figure 2 Mean alpha diversity over time, by circumcision status. The mean alpha diversity measures are shown over time (black lines) with the 95% confidence interval shown in the shaded areas around the mean line. Alpha diversity measures over time are stratified by circumcision status, in which pink represents men who are uncircumcised and blue represents men who are circumcised.

Supplementary Figure 3 Fractional polynomial fitted plot with 95% confidence intervals for alpha diversity measures over time, stratified by Nugent Score Category. The fitted line plots show the change in **(A)** Shannon Diversity Index and **(B)** Richness over time in months (x-axis), stratified by circumcision status and female partner BV status.
