# Supplementary figures and images for: Longitudinal Changes in the Composition of the Penile Microbiome Are Associated With Circumcision Status, HIV and HSV-2 Status, Sexual Practices, and Female Partner Microbiome Composition
Source: Front Cell Infect Microbiol. 2022 Jul 5;12:916437. doi: 10.3389/fcimb.2022.916437 (PMC9294230; doi:10.3389/fcimb.2022.916437)

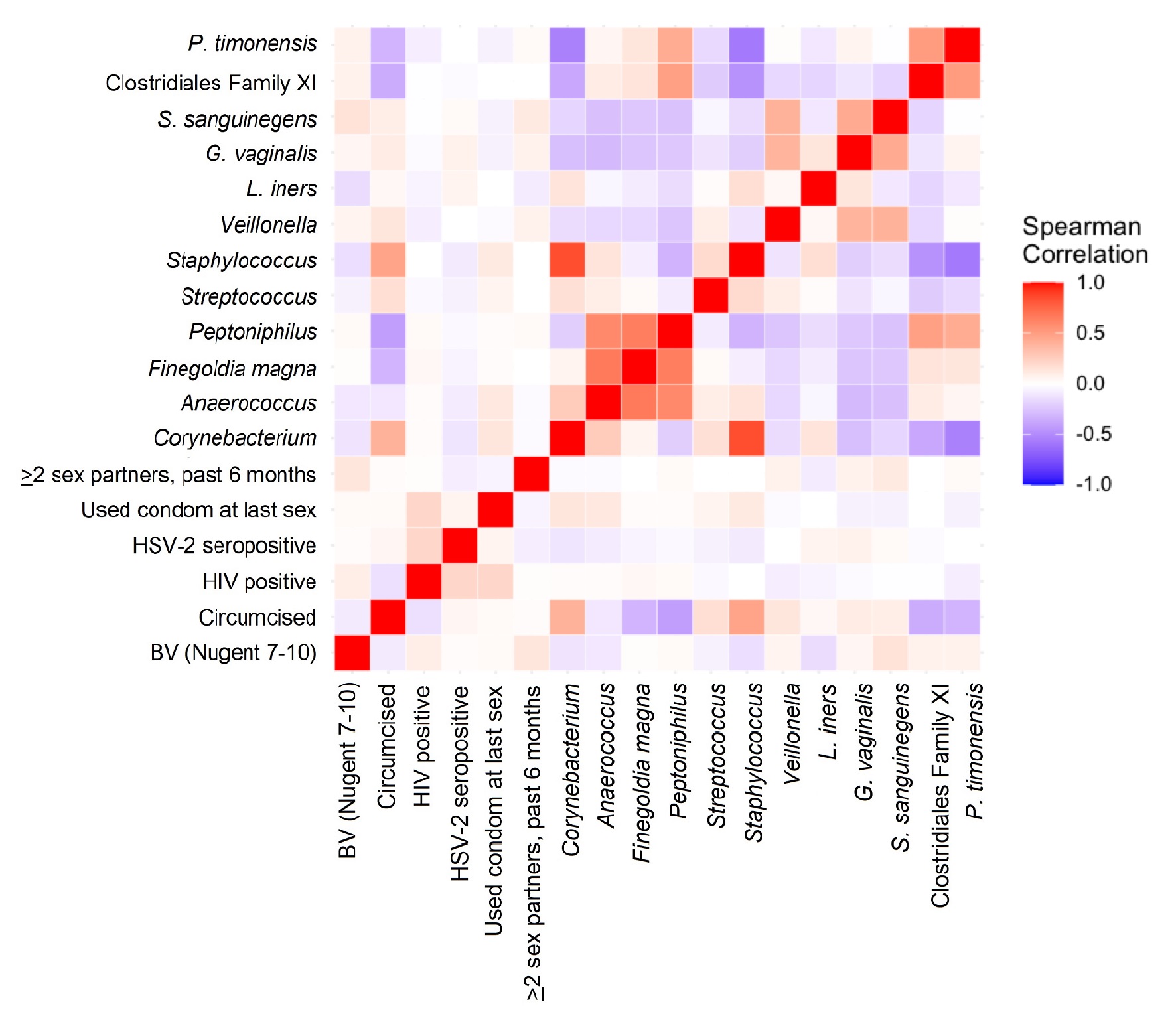

Supplement: Supplementary Figure 1 — Correlation heatmap showing the Spearman correlations among twelve taxa with highest relative abundance and selected covariates of importance. Spearman rank correlation heatmap of the top 12 penile taxa with greatest relative abundance, and selected covariates. Relative abundance is center log ratio transformed prior to correlation estimation. The correlation heat map represents the direction and magnitude of the Spearman rank correlation between the taxa and covariates. Negative correlations are shaded in blue and positive correlations in red, with deeper intensity representing the magnitude of the correlation. Taxa are represented at the genus level, except where species is noted, for the following: Prevotella timonensis, Sneathia sanguinegens, Gardnerella vaginalis, and Lactobacillus iners. [file Image_1.jpeg]

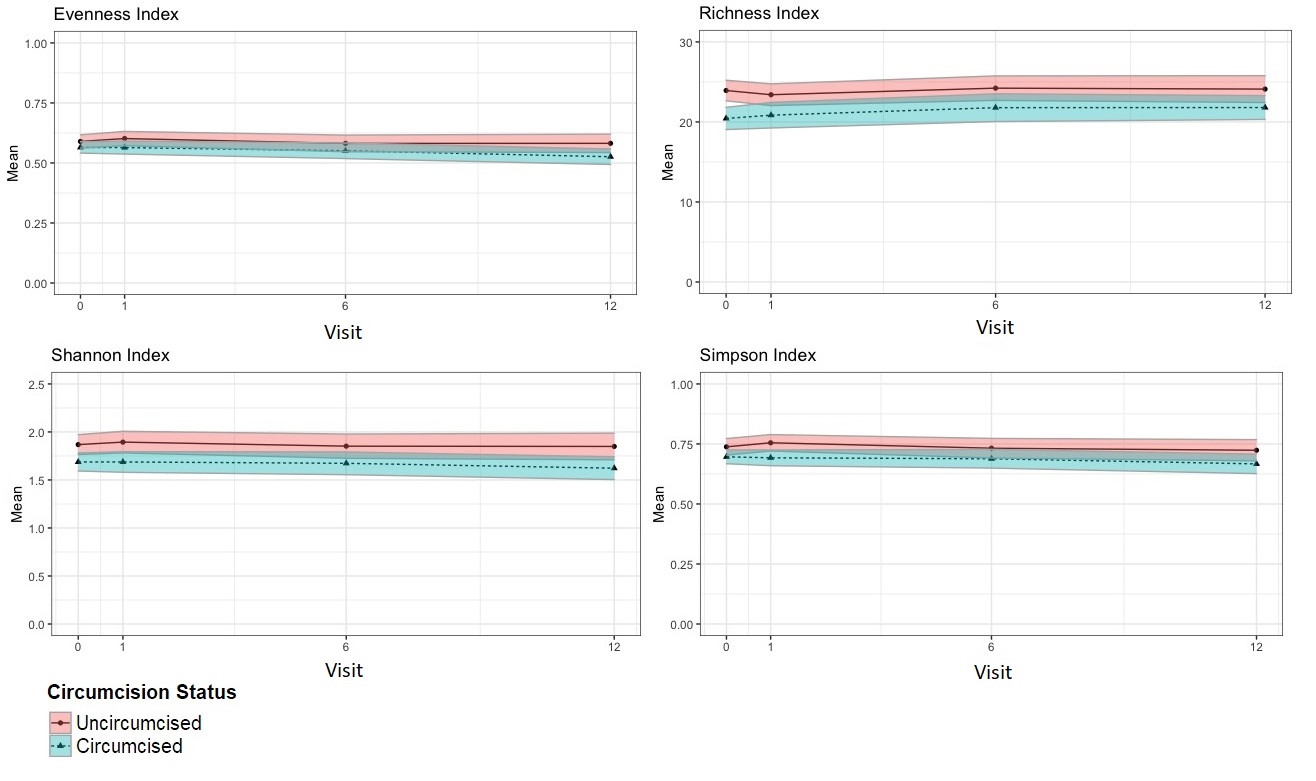

Supplement: Supplementary Figure 2 — Mean alpha diversity over time, by circumcision status. The mean alpha diversity measures are shown over time (black lines) with the 95% confidence interval shown in the shaded areas around the mean line. Alpha diversity measures over time are stratified by circumcision status, in which pink represents men who are uncircumcised and blue represents men who are circumcised. [file Image_2.jpeg]

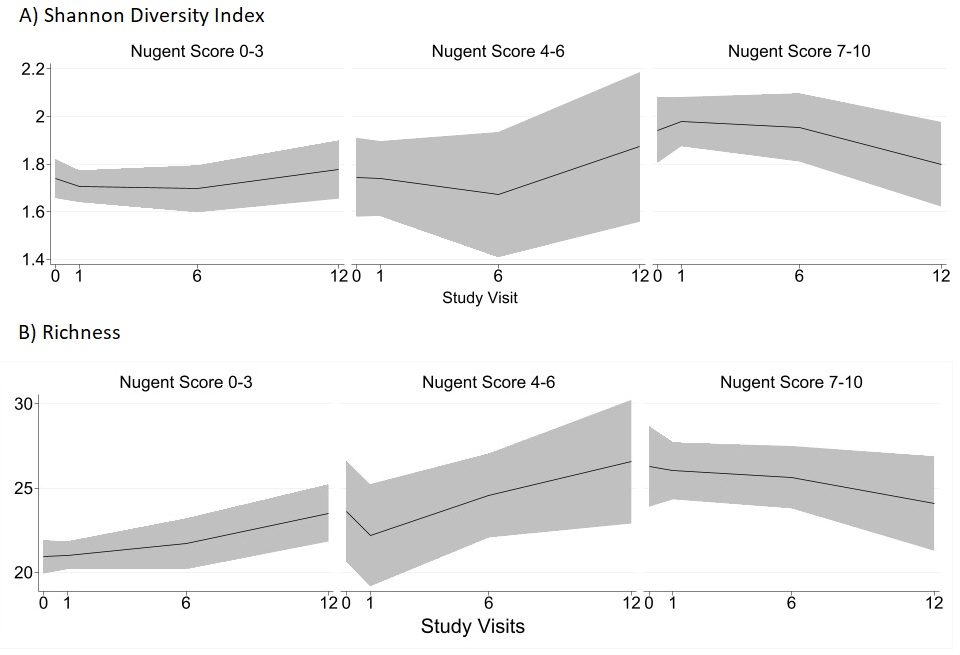

Supplement: Supplementary Figure 3 — Fractional polynomial fitted plot with 95% confidence intervals for alpha diversity measures over time, stratified by Nugent Score Category. The fitted line plots show the change in (A) Shannon Diversity Index and (B) Richness over time in months (x-axis), stratified by circumcision status and female partner BV status. [file Image_3.jpeg]
