# Supplementary material for: Longitudinal Changes in the Composition of the Penile Microbiome Are Associated With Circumcision Status, HIV and HSV-2 Status, Sexual Practices, and Female Partner Microbiome Composition
Source: Front Cell Infect Microbiol. 2022 Jul 5;12:916437. doi: 10.3389/fcimb.2022.916437 (PMC9294230; doi:10.3389/fcimb.2022.916437)
Supplement: Supplementary file 4 [file Table_1.docx]

**Supplemental Table 1. Results of mixed effects multinomial modeling*: Time-adjusted univariate odds ratio and p-value of Penile Meatal Community Type.**

|  | CT-1 Mixed  vs. CT-2  OR (p-value) | CT-3  *Streptococcus* dominant vs. CT-2  OR (p-value) | CT-4  *S. sanguinegens* dominant vs. CT-2  OR (p-value) | CT-5  *Finegoldia/*  *Anaerococcus* dominant vs. CT-2  OR (p-value) | CT-6  *L. iners* dominant vs. CT-2  OR (p-value) | CT-7  *Clostridiales/*  *Peptoniphilus* dominant vs. CT-2  OR (p-value) |
| --- | --- | --- | --- | --- | --- | --- |
| Men’s age in years, continuous | 1.03 (p=0.515) | 0.96 (p=0.416) | 0.93 (0.112) | 1.04 (p=0.391) | 0.98 (p=0.737) | 1.04 (p=0.448) |
| Educational attainment At least some high school or more (vs. primary or less) | 0.92 (p=0.785) | 1.33 (p=0.476) | 0.67 (p=0.248) | 0.59 (p=0.118) | **0.50 (p=0.073)** | **0.48 (p=0.052)** |
| Currently employed  (vs. unemployed) | 1.27 (p=0.440) | 1.69 (p=0.230) | 1.58 (p=0.223) | 1.06 (p=0.872) | 0.65 (p=0.258) | 0.80 (p=0.566) |
| Circumcised  (vs. uncircumcised) | **0.53 (p=0.056)** | 0.57 (p=0.188) | **0.45 (p=0.030)** | **0.04 (p<0.001)** | **0.25 (p<0.001)** | **0.02 (p<0.001)** |
| HIV positive  (vs. negative) | 0.66 (p=0.375) | 0.58 (p=0.386) | 0.75 (p=0.592) | 1.29 (p=0.591) | 0.70 (=0.548) | 0.76 (p=0.633) |
| HSV-2 positive  (vs. negative) | 0.85 (p=0.588) | 1.03 (p=0.937) | 0.98 (p=0.959) | 1.01 (p=0.963) | 1.01 (p=0.969) | 1.39 (p=0.373) |
| Condom use sometimes or always (vs. never) | 0.66 (p=0.142) | 1.07 (p=0.847) | 0.61 (p=0.135) | 0.87 (p=0.652) | **0.48 (p=0.053)** | **0.46 (p=0.034)** |
| Condom used at last sexual intercourse | 0.58 (p=0.114) | 0.72 (p=0.460) | **0.36 (p=0.021)** | 0.87 (p=0.697) | 0.54 (p=0.176) | 0.49 (p=0.114) |
| Two or more sex partners in past 6 months (vs. one) | 1.06 (p=0.858) | 0.97 (p=0.942) | 1.83 (p=0.107) | 1.11 (p=0.783) | **0.33 (p=0.068)** | 1.40 (p=0.426) |
| Days since last sex  (vs. 0-2 days)  3-6 days  7 or more days | 0.61 (p=0.156)  0.64 (p=0.166) | 1.18 (p=0.713)  1.13 (p=0.773) | 0.89 (p=0.776)  0.77 (p=0.490) | 0.92 (p=0.816)  1.14 (p=0.699) | 0.56 (p=0.204)  0.71 (p=0.395) | 0.87 (p=0.743)  0.77 (p=0.527) |
| Washed penis more than one hour after last sex (vs. <=1 hour) | 1.33 (p=0.370) | 1.07 (p=0.863) | 0.87 (p=0.683) | **2.15 (p=0.031)** | **2.13 (p=0.081)** | **2.20 (p=0.060)** |
| Received antibiotics at study visit | 0.73 (p=0.525) | 0.77 (p=0.672) | 0.53 (p=0.272) | 0.55 (p=0.272) | **0.15 (p=0.083)** | 0.82 (p=0.737) |
| Visit (vs. Baseline)  1 Month  6 Months  12 Months | 1.21 (p=0.554)  **2.51 (**p=**0.011)**  **2.05 (**p=**0.040)** | 0.84 (p=0.693)  1.44 (p=0.449)  1.20 (p=0.692) | 0.97 (p=0.932)  1.31 (p=0.520)  0.74 (p=0.492) | 1.03 (p=0.943)  1.87 (p=0.106)  1.08 (p=0.843) | 1.29 (p=0.556)  **2.73 (**p=**0.030)**  1.05 (p=0.920) | 0.78 (p=0.555)  1.45 (p=0.419)  1.12 (p=0.805) |
| *Female partner characteristics* |  |  |  |  |  |  |
| Age in years (continuous) | 1.01 (p=0.873) | 0.97 (p=0.505) | 0.96 (p=0.329) | 1.07 (p=0.103) | 0.97 (p=0.524) | 1.04 (p=0.424) |
| Nugent BV  (7-10 score vs. 0-6 score) | 1.14 (p=0.705) | 0.94 (p=0.898) | 1.51 (p=0.273) | 0.79 (p=0.543) | 0.59 (p=0.281) | 1.82 (p=0.136) |
| HIV positive  (vs. negative) | 0.72 (p=0.529) | 0.54 (p=0.406) | 1.18 (p=0.772) | 1.70 (p=0.307) | 1.21 (p=0.760) | 2.03 (p=0.207) |
| HSV-2 positive  (vs. negative) | 1.68 (0.112) | 0.95 (p=0.896) | 1.56 (p=0.213) | 1.69 (p=0.130) | 1.56 (p=0.265) | 1.74 (p=0.158) |
| Community State Type  (vs. CST-1)  CST-III  CST-IV | 1.80 (p=0.240)  2.02 (p=0.162) | 0.60 (p=0.377)  0.65 (p=0.450) | 1.55 (p=0.447)  2.03 (p=0.222) | 0.86 (p=0.759)  0.82 (p=0.693) | 1.21 (p=0.743)  0.71 (p=0.582) | 3.40 (p=0.147)  **5.32 (p=0.045)** |

*Reference category is Community Type 2 (CT-2; *Corynebacterium* dominant).

OR = Odds Ratio.

**Supplemental Table 2. Results of Multivariable Mixed Effects Modeling of 12 Individual Taxa with Highest Mean Relative Abundance.**

| **Taxa**  **Covariates, β (p-value)** | Clostridiales Family XI | *S. sanguinegens* | *G. vaginalis* | *L. iners* | *Veillonella* | *Staphylococcus* | *Streptococcus* | *Peptoniphilus* | *Finegoldia magna* | *Anaerococcus* | *Corynebacterium* | *P. timonensis* |
| --- | --- | --- | --- | --- | --- | --- | --- | --- | --- | --- | --- | --- |
| Circumcised (vs. uncircumcised) | -1.97  (<0.001) |  |  |  |  | 2.55  (<0.001) | 0.92  (0.002) | -1.52  (<0.001) | -1.25  (<0.001) | -0.33  (0.037) | 1.88  (<0.001) | -2.13  (<0.001) |
| Nugent BV  (7-10 score vs. 0-6 score) |  | 0.66  (0.029) |  |  | -0.22  (0.682) | -0.51  (0.027) |  |  |  | -0.34  (0.025) |  |  |
| HIV Positive (vs. negative) |  |  |  |  |  |  |  |  |  |  |  | -1.04  (0.026) |
| HSV-2 Seropositive (vs. seronegative) |  |  |  |  |  | -0.58  (0.015) |  |  |  |  |  |  |
| Condom used at last sexual intercourse |  |  |  |  |  | 0.58  (0.027) |  |  |  | 0.36  (0.037) |  |  |
| Condom use sometimes or always (vs. never) |  |  | -0.51  (0.043) |  |  |  |  |  |  |  | 0.42  (0.020) |  |
| Two or more sex partners in past 6 months (vs. one) |  | 0.69  (0.043) | 0.78  (0.010) |  |  |  |  |  |  |  |  |  |
| Community State Type  (vs. CST-1) |  |  |  |  |  |  |  |  |  |  |  |  |
| CST-III | 0.35  (0.713) | -0.97  (0.414) |  | 6.75  (<0.001) |  | -0.39  (0.665) |  | 0.71  (0.273) |  | -0.18  (0.758) |  |  |
| CST-IV | 0.92  (0.321) | 0.30  (0.796) |  | -0.01  (0.993) |  | -0.97  (0.270) |  | 1.07  (0.093) |  | 0.31  (0.594) |  |  |
| Days since last sex (vs. 0-2 days) |  |  |  |  |  |  |  |  |  |  |  |  |
| 3-6 days |  |  | -0.81  (0.003) |  |  |  |  |  |  |  | 0.50 (0.007) |  |
| 7 or more |  |  | -1.04  (<0.001) |  |  |  |  |  |  |  | 0.41  (0.031) |  |
| Age in Years (continuous) |  | -0.11  (0.007) | -0.09  (0.006) |  |  |  |  |  |  | 0.04  (0.049) |  |  |
| At least some high school or more (vs. primary or less) |  |  | -0.56  (0.036) |  |  |  | 0.74  (0.012) |  |  |  | 0.49  (0.035) |  |
| Received antibiotics at study visit or reported |  |  |  |  | 1.06  (0.028) | 0.78  (0.029) |  |  |  |  |  |  |
| Study Visit (vs. Baseline) |  |  |  |  |  |  |  |  |  |  |  |  |
| 1 Month | -0.10  (0.617) | -0.07  (0.792) | -0.45  (0.102) | -0.02  (0.924) | -0.25  (0.450) | 0.08  (0.703) | -0.03  (0.913) | 0.03  (0.834) | -0.06  (0.717) | -0.04  (0.800) | -0.09  (0.572) | -0.21  (0.366) |
| 6 Months | -0.24  (0.309) | -0.59  (0.053) | -0.12  (0.685) | 0.47  (0.046) | -0.98  (0.007) | -0.16  (0.49) | -0.25  (0.389) | -0.46  (0.004) | -0.33  (0.063) | -0.25  (0.110) | -0.23  (0.205) | -0.11  (0.661) |
| 12 Months | -0.13  (0.612) | -0.50  (0.135) | -0.14  (0.662) | 0.01  (0.096) | -0.13  (0.737) | -0.45  (0.077) | -0.50  (0.119) | -0.76  (<0.001) | -0.81  (<0.001) | -0.73  (<0.001) | -0.65  (0.001) | -0.21  (0.468) |
| Interaction of BV status with time |  |  |  |  |  |  |  |  |  |  |  |  |
| Nugent BV X Visit 1 Month |  |  |  |  | 0.30  (0.677) |  |  |  |  |  |  |  |
| Nugent BV X Visit 6 Months |  |  |  |  | 2.14  (0.006) |  |  |  |  |  |  |  |
| Nugent BV X Visit 12 Months |  |  |  |  | 0.63  (0.446) |  |  |  |  |  |  |  |

**Supplemental Table 3. Results of Mixed Effects Linear Regression: Factors associated with Penile Meatal Microbiome Shannon Diversity Index and Richness Over time.**

|  | Shannon Diversity Index | | Richness | |
| --- | --- | --- | --- | --- |
| Variables | Univariate^+^  Coefficient (95%CI) | Multivariable Adjusted^#^, N=221 subjects with 654 observations  Coefficient (95%CI) | Univariate  Coefficient (95%CI) | Multivariable Adjusted^#^, N=221 subjects with 654 observations  Coefficient (95% CI) |
| Circumcised  (vs. uncircumcised) | -0.205 (-0.307 - -0.102)^a^ | -0.227 (-0.327 - -0.126)^a^ | -2.90 (-4.35 - -1.45)^a^ | -2.92 (-4.33 - -1.51)^a^ |
| Female partner has BV  (vs. Nugent score 0-6) | 0.171 (0.075 – 0.266)^a^ | 0.110 (0.006 – 0.214)^b^ | 2.51 (1.15 – 3.86)^a^ | 1.25 (-0.201 – 2.71)^c^ |
| Education: Some high school or more (vs. primary or less) |  |  | -1.80 (-3.32 - -0.28)^b^ | -1.80 (-3.21 - -0.38)^b^ |
| Currently Employed  (vs. unemployed) | 0.128 (0.034 – 0.222)^a^ | 0.198 (0.102 – 0.293)^a^ | 2.01 (0.71 – 3.30)^a^ | 2.60 (1.28 – 3.93)^a^ |
| Condom use frequency past 6 months: “Sometimes” or “Always” vs. “Never” | -0.101 (-0.189 - -0.014)^b^ | -0.094 (-0.183 - -0.006)^b^ | -1.45 (-2.67 - -0.35)^b^ | -1.48 (-2.71 - -0.25)^b^ |
| Female sex partner’s community state type (vs. CST-I *L. crispatus* dominated)  III *L. iners* dominated  IV *G. vaginalis* dominated | 0.130 (-0.016 – 0.276)^c^  0.256 (0.108 – 0.504)^a^ | 0.121 (-0.020 – 0.261)^c^  0.205 (0.056 – 0.355)^a^ | 2.59 (0.57 – 4.61)^b^  5.26 (3.12 – 7.20)^a^ | 2.34 (0.40 – 4.28)^b^  4.49 (2.43 – 6.56)^a^ |
| Days since last sexual intercourse (vs. 0-2 days)  3 – 6 days  7 or more days | -0.118 (-0.217 - -0.020)^b^  -0.102 (-0.195 - -0.008)^b^ | -0.115 (-0.215 - -0.015)^b^  -0.114 (-0.210 - -0.018)^b^ | -2.39 (-3.73 - -1.03)^a^  -1.92 (-3.20 - -0.634)^a^ | -2.51 (-3.90 - -1.13)^a^  -1.73 (-3.05 - -0.40)^b^ |
| Female partner HSV-2 positive (vs. negative) | 0.088 (-0.016 – 0.192)^c^ |  | 1.54 (0.043 – 3.04)^b^ | NS |
| Study Visit (vs. Baseline)  1 Month  6 Months  12 Months | 0.005 (-0.085 – 0.094)  -0.021 (-0.115 – 0.073)  -0.043 (-0.139 – 0.054) | 0.038 (-0.052 – 0.128)  0.009 (-0.088 – 0.106)  0.056 (-0.049 – 0.161) | -0.141 (-1.35 – 1.07)  0.713 (-0.564 – 1.99)  0.912 (-0.401 – 2.23) | 0.26 (-0.98 – 1.50)  1.11 (-0.23 – 2.45)  2.11 (0.66 – 3.56)^a^ |
| *Male characteristics with p>0.10 in univariate analysis* |  |  |  |  |
| Two or more sex partners past 6 months (vs. one) | -0.011 (-0.117 – 0.096) |  | -0.358 (-1.87 – 1.15) |  |
| Age in years (continuous) | 0.004 (-0.009-0.018) |  | -0.072 (-0.265- 0.122) |  |
| Education: Some high school or more (vs. primary or less) | -0.041 (-0.149 – 0.067) |  |  |  |
| Washed penis more than one hour after last sex (vs. <=1 hour) | 0.012 (-0.085 – 0.108) |  | -0.536 (-1.87 – 0.803) |  |
| HIV positive (vs. negative) | -0.032 (-0.195 – 0.130) |  | 0.062 (-2.24 – 2.37) |  |
| HSV-2 positive (vs. negative) | 0.006 (-0.095 – 0.107) |  | 0.101 (-1.33 – 1.53) |  |
| Condom used at last sexual intercourse (vs. condom not used) | -0.009 (-0.119 – 0.100) |  | -0.161 (-1.69 – 1.37) |  |
| Received antibiotics at a study visit | -0.027 (-0.177 – 0.123) |  | -0.215 (-2.27 – 1.84) |  |
| *Female characteristics with p>0.10 in univariate analysis* |  |  |  |  |
| Age in years (continuous) | 0.006 (-0.007 – 0.019) |  | -0.025 (-0.213 –0.164) |  |
| Female partner HIV positive (vs. negative) | 0.033 (-0.125 – 0.192) |  | 1.79 (-0.483 – 4.07) |  |

^+^ Univariate associations are time-adjusted.

# Multivariable models are simultaneously adjusted for all variables presented

^a^p<0.01, ^b^p<0.05, ^c^p<0.10

**Supplemental Table 4. Results of multinomial mixed effects regression: factors associated with stability of Bray Curtis similarity measure over time.**

|  | Less Stable (<41% similarity between visits)  vs.  Stable (50-<70% similarity between visits)  Adjusted OR [95% CI] | More Stable (>70% similarity between visits)  vs.  Stable (50-<70% similarity between visits)  OR [95% CI] |
| --- | --- | --- |
| *Male Characteristics* |  |  |
| Age in years (continuous) | 0.92 [0.84 – 1.01]^c^ | 0.92 [0.84 – 1.01]^c^ |
| Currently employed (vs. unemployed) | 0.42 [0.18 – 0.97]^b^ |  |
| HSV-2 seropositive (vs. negative) | 2.99 [1.36 – 6.56]^a^ |  |
| Antibiotic use | 0.14 [0.02 – 1.19]^c^ |  |
| Condom use “always” or “sometimes” vs. “never” |  | 0.50 [0.23 – 1.11]^c^ |
| Visit | 0.96 [0.85 – 1.09] | 0.99 [0.88 – 1.12] |

Model is simultaneously adjusted for all variables presented

aOR = Odds ratio; 95% CI = 95% Confidence Interval

^a^ p<0.01; ^b^ p<0.05; ^c^ 0.05<p<0.1
